# Diversification of Bacterial Community Composition along a Temperature Gradient at a Thermal Spring

**DOI:** 10.1264/jsme2.ME11350

**Published:** 2012-05-17

**Authors:** R. Craig Everroad, Hiroyo Otaki, Katsumi Matsuura, Shin Haruta

**Affiliations:** 1 Department of Biological Sciences, Graduate School of Science and Engineering, Tokyo Metropolitan University, 1–1 Minami-Osawa, Hachioji-shi, Tokyo 192–0397, Japan

**Keywords:** biogeography, sulfur oxidizing bacteria, photosynthetic bacteria, thermophile, *Aquificales*

## Abstract

To better understand the biogeography and relationship between temperature and community structure within microbial mats, the bacterial diversity of mats at a slightly alkaline, sulfide-containing hot spring was explored. Microbial mats that developed at temperatures between 75–52°C were collected from an area of approximately 1 m^2^ in Nakabusa, Nagano, Japan. Bacterial 16S rRNA genes from these samples were examined by terminal restriction fragment length polymorphism (T-RFLP) and clone library analysis. T-RFLP profiles revealed 66 unique fragments (T-RFs). Based on total T-RFs observed in environmental profiles and clone libraries, a temperature effect on diversity was determined, with complexity in the community increasing as temperature decreased. The T-RF pattern indicated four distinct community assemblages related to temperature. Members of the *Aquificales* and particularly the sulfuroxidizing bacterium *Sulfurihydrogenibium* were present at all temperatures and were the dominant component of mats taken at 75–67°C. Sulfide oxidation, which persisted throughout the temperature gradient, was the presumed dominant pathway of primary production above 67°C. As temperature decreased, successive additions of anoxygenic and oxygenic phototrophs increased primary productivity, allowing for diversification of the community.

Understanding how the environment shapes microbial communities is a current challenge in microbial ecology (60, 65). Microbes play important roles in structuring ecosystems and in geochemical cycles (4, 8, 37); however, because microorganisms are extremely small, numerically abundant, markedly diverse and globally dispersed, an understanding of their biogeography has proven difficult (31, 49, 76), if it exists at all (15). Recent investigations have explored whether the rules that structure communities for larger organisms also hold true for microbes, *e.g.*, Bryant *et al.* (6) and Green and Bohannan (20). In particular, identifying environmental parameters that shape the distribution of microbial taxa and communities has recently received much attention on continental (14, 51, 63), regional (6, 47, 74), local (13, 23, 61, 70, 82) and millimeter scales (44, 48, 83).

Microbial mats that develop in thermal springs have been proposed as suitable habitats to explore patterns of microbial abundance (52). The distribution of bacteria along hot spring outflows and the effects of temperature on these transitions on meter to kilometer scales has been reported for terrestrial hot springs and a subsurface geothermal water stream (13, 36, 52, 72). On such scales, several factors, not just temperature but also chemical components of the water, pH and distance from the source may also affect the succession of microbes. In contrast, clear temperature gradients of spring water in a limited area are observed at Nakabusa hot spring (73), which is a sulfidic, slightly alkaline (pH ranging from 8.3 to 8.9; sulfide concentration approximately 0.1 mM) geothermal spring found in Nagano Prefecture, Japan. The outflow emerges from seams in a sediment-control dam wall and several types of microbial mats develop on this wall. Differences in the volume of the individual outflow(s) create a spatially and temporally heterogeneous environment with areas within a 1 m radius ranging from 75°C to 50°C. These microbial mats are an ideal location to clarify the bacterial distribution along a temperature gradient.

Nakabusa hot spring is one of several well-studied thermal springs worldwide. *Roseiflexus castenholzii*, a filamentous photosynthetic bacterium lacking chlorosomes, was isolated from microbial mats on the dam wall at Nakabusa (27). However, only a few studies have explicitly explored the microbial diversity found here. Kubo *et al.* (43) determined the community members contributing to sulfur metabolism from 65°C at Nakabusa hot spring using clone library analyses. In a pioneering molecular ecological study in Nakabusa, Nakagawa and Fukui (57) surveyed microbial communities of mats that had developed at 6 temperatures (76 to 48°C) from 2 sites using denaturing gradient gel electrophoresis (DGGE) targeting 16S rRNA genes. They detected approximately 16 bacterial and archaeal taxa. The DGGE profiles indicated the existence of a major break in community composition between 60 and 66°C. Using DGGE, Kubo *et al.* (43) also determined clear differences between community members from 65 and 75°C. These previous studies, however, did not address quantitative questions of temperature-based changes in microbial communities.

The aim of the present study was to expand the current understanding of how temperature structures microbial communities at Nakabusa hot spring by sampling more temperatures in a smaller area than had been previously attempted (43, 57). To address this question, a broad-scale survey of molecular diversity of bacteria across a range of temperatures from 75°C to 52°C using terminal restriction fragment length polymorphism (T-RFLP) and sequence analysis of clone libraries of 16S rRNA genes amplified from environmental DNA was undertaken. Using statistical and phylogenetic analyses, this work quantitatively describes the patterns of individual taxa within the bacterial community structure and discusses the effects of temperature changes on the overall community structure and metabolism.

## Materials and Methods

### Sampling site and sampling

Nakabusa hot spring is located in Nagano Prefecture in Japan (36°23′15″ N, 137°45′00″ E, 1,500 m elevation). The hot spring is slightly alkaline, with a pH of 8.6±0.3. Mat samples were collected aseptically from various points on the dam wall on July 5th, 2008 at temperatures ranging from 75 to 52°C (Fig. S1). Mat samples were brought to the laboratory at room temperature in either 15 mL or 50 mL polypropylene tubes filled with hot spring water and without headspace. Once in the laboratory (about 4 h after sampling), subsamples were homogenized using a Polytron homogenizer (Kinematica, Littau-Lucerne, Switzerland) and aliquots of the mat samples ranging from 0.06 to 0.34 g were placed in 1.5 mL microcentrifuge tubes and frozen at −20°C until DNA extraction.

### DNA extraction, PCR and cloning

Bulk DNA from mat samples was isolated using a modified chloroform phenol extraction protocol as described in (43). Briefly, samples were disrupted with freeze/thaw and bead-beating steps, and then further lysed using lysozyme and proteinase K. After adding NaCl and hexadecyl-trimethyl-ammonium bromide (CTAB) to 0.95 M and 1% w/v respectively, genomic DNA was extracted by successive chloroform-isoamyl alcohol and phenol-chloroformisoamyl alcohol steps and precipitated with isopropanol. Bacterial 16S rRNA genes were PCR-amplified for both clone libraries and T-RFLP analysis using the universal primers Ba27f and Ba907R (55, 78). PCR was performed in 25 μL volumes using ExTaq (Takara, Otsu, Japan). The manufacturer’s general reaction protocol was followed with 1 μL extracted genomic DNA and 0.5 μM of each primer was added. The PCR protocol was as follows: 94°C for 3 min followed by 25 cycles of 94°C for 30 s, 52°C for 45 s, 72°C for 90 s, and a final elongation step at 72°C for 5 min. PCR products were verified on ethidium bromide-stained 1.5% (w/v) agarose gels. PCR products were gel purified in 50 μL sterile water using the QIAquick gel extraction kit (Qiagen, Hilden, Germany).

Clone libraries of selected samples (from 69, 63, 58, and 52°C) were constructed by ligating purified PCR products into the DynaExpress TA PCR cloning kit vector (BioDynamics, Tokyo, Japan). *Escherichia coli* JM109 competent cells were transformed chemically and grown overnight. PCR was performed as above but with 23 cycles on positive clones directly by touching the colony with a pipette tip and then dipping the tip into prepared PCR mix. Vector primer pairs were used according to the manufacturer’s instructions.

### T-RFLP analysis

For T-RFLP analysis, the forward primer Ba27F was labeled with 6-carboxyfluorescein (FAM) at the 5′ end. For each DNA sample, PCR was performed as described above. Then, 2–4 μL of purified PCR product, or approximately 100 ng DNA from either environmental samples or clones were digested with 5 U *Msp*I at 37°C for at least 16 h. For each digest, an aliquot of 0.3 μL (for environmental DNA) or 0.15 μL (for clones) was added to 15 μL 40:1 HiDi formamide: internal size standard (GeneScan 600 LIZ size standard; Applied Biosystems, Carlsbad, CA, USA). Labeled terminal-restriction fragments were denatured at 92°C for 1 min and immediately chilled on ice. Fragment profiles were determined using an ABI 3130xl capillary DNA sequencer in GeneScan mode. For environmental samples, fragments with a relative fluorescence signal >50 were retained for further analyses. To normalize T-RF profiles, the “standardization of DNA quantity between replicate profiles” approach described by Dunbar *et al.* (10) was used. Briefly, total florescence was calculated for each sample and a correction factor was applied so that all profiles had a total fluorescence equaling that of the sample with the lowest total fluorescence. After removing peaks that were corrected to <50 fluorescence units, the process was repeated three more times until no further peaks disappeared. T-RFLP data were converted to binary (presence/absence) matrices and within-sample proportional signal heights for summary and statistical analyses (7).

To identify individual T-RFs, clone libraries of samples from 75, 66, 60, and 56°C were constructed and analyzed in addition to the samples from 69, 63, 58, and 52°C as described above.

### Sequencing, phylogenetic and cluster analyses

Partial 16S rRNA gene sequences of clones were obtained after PCR amplifications from *E. coli* colonies using the BigDye Terminator v3.1 cycle sequencing kit (Applied Biosystems) with vector primers as sequencing primers. Sequences were run on an Applied Biosystems 3130xl capillary sequencer. Fragments were sequenced directly in both directions. The sequence data were visually edited and assembled using BioEdit Sequence Alignment Editor v.7.0.9.0 (24). Assembled sequences were screened for chimeras using Bellephron and Mallard (3, 33) and manually as needed by comparing partial sequence Basic Local Alignment Search Tool (BLAST) results (86) at the National Center for Biotechnology Information (http://www.ncbi.nlm.nih.gov). Edited sequences were putatively identified using BLAST, with taxonomic affiliations crudely assigned by sequence identity to cultured isolates.

For phylogenetic analysis of *Sulfurihydrogenibium*-like sequences, sequence alignments were made with several other related taxa using ClustalX 2.0.5 with default gap penalties (30, 46). Alignments were visually inspected and gaps were excluded for phylogenetic analyses. Missing data were coded as missing. Maximum likelihood analyses were performed using the PhyML 3.0 on-line webserver (22). Likelihood analyses used the SPR and NNI tree searching option for 5 starting BIONJ trees (18) and were bootstrapped 100 times. Bayesian analysis was performed with MrBayes 3.1.2 (34, 69). Two independent runs of four chains (one cold) of Metropolis-coupled Markov chain Monte Carlo generations were run for 1×10^6^ generations with trees sampled every 100 generations. Of these 10,000 sample trees, 2,500 were discarded as burn-in. Posterior probabilities were determined from a majority rule consensus tree calculated from the remaining trees. The nucleotide substitution model for both analyses was set to conform to the general time reversible model with gamma distribution and proportion of invariable sites (GTR+I+Γ) as indicated by AIC in jModeltest ver.0.0.1 (64). Parameter values were estimated by PHYML for the likelihood tree, and the default priors were set for Bayesian analysis.

Unweighted pair group method with arithmetic mean (UPGMA) cluster analysis was performed on the raw binary T-RF matrix using DendroUPGMA online (http://genomes.urv.cat/UPGMA/index.php) (17). Similarity coefficients and a distance matrix for the UPGMA dendrogram were calculated using the Dice similarity coefficient (9). For clone libraries, the Shannon diversity index (*H′*) and rarefaction curves were calculated using FastGroupII (71, 84).

### Nucleotide sequence accession numbers

The nucleotide sequences reported in this study were deposited in the DDBJ/EMBL/GenBank database with the following accession numbers: JF826964–JF827017.

## Results

### T-RFLP, clone library and sequencing results

**Fig. 1:**
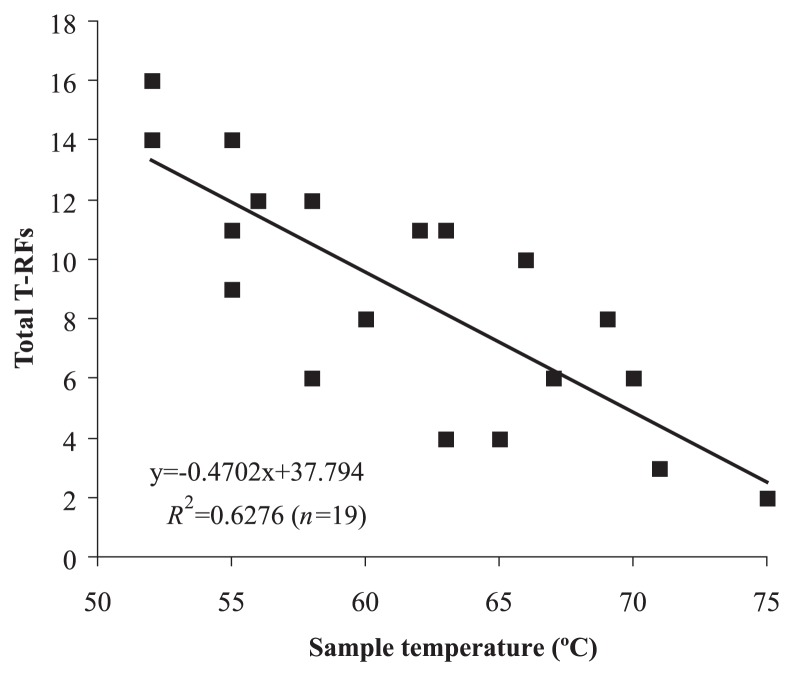
Regression between temperature and total number of T-RFs for each sample collected. T-RF signals were normalized by sample to the lowest sample value to correct for variations in loading between samples and for an unbiased estimate of richness. Temperature has a significant effect on the number of T-RFs observed (*R*^2^=0.6276, *t*=6.97 (17); *P*<0.0001). Raw values also showed a similar relationship, but with a lower *R*^2^ value (0.39).

**Fig. 2:**
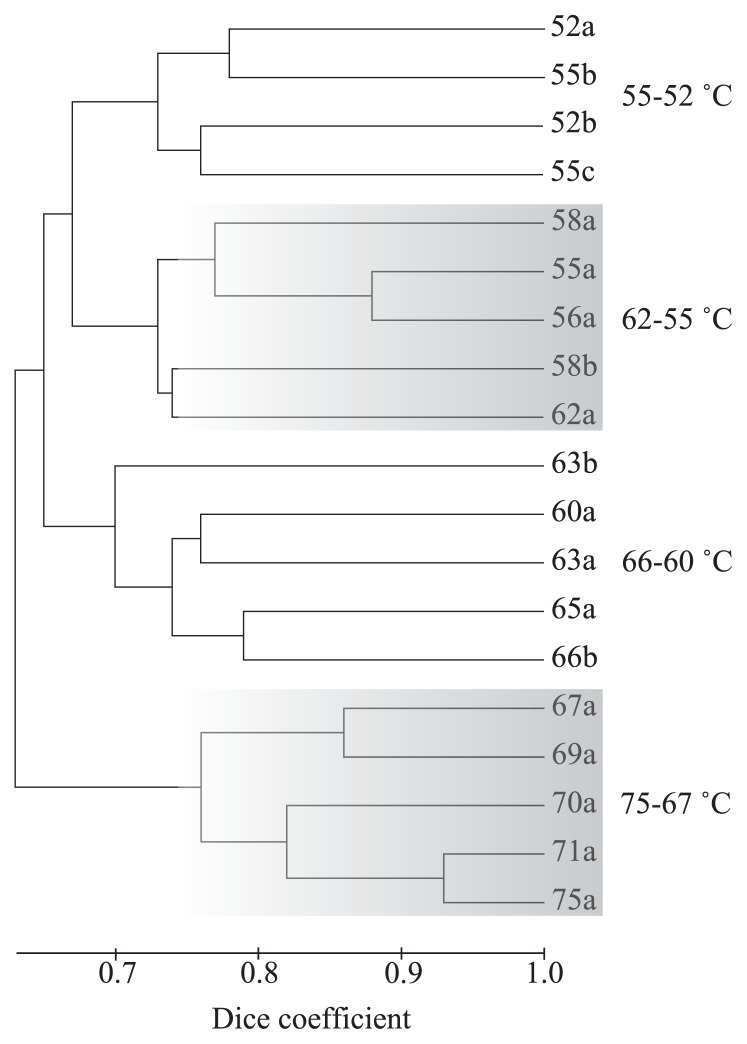
UPGMA cluster analysis of T-RF patterns for all samples analyzed. Sample number indicates temperature (°C).

In all, 66 unique T-RFs were recovered from 19 environmental T-RFLP profiles sampled from 14 temperatures (75, 71, 70, 69, 67, 66, 65, 63, 62, 60, 58, 56, 55 and 52°C). The environmental T-RFs and their distributions are provided in Table S1. A significant inverse relationship between the sample temperature and the number of T-RFs was detected by linear regression, as shown in Fig. 1. As the sample temperature decreased the number of T-RFs increased. The UPGMA dendrogram using the raw binary T-RF matrix revealed four partitions related to temperature, with each group ranging from 75–67°C, 66–60°C, 62–55°C and 55–52°C (Fig. 2). Distance matrices for UPGMA calculated using the Jaccard similarity coefficient and Pearson’s *r* recovered near identical patterns (data not shown). These partitions could also be related to the shape and color of the mats, *i.e.*, grey or white streamers at the highest temperatures, olive green mats of a few millimeters at around 65°C, and thick dark-green mats below 62°C. Visually, mats at 62–55°C were barely distinguishable from those at 55–52°C, but the dark-green mats, a part of which sometimes turned yellowish, tended to thicken as the temperature decreased.

The composition of clone libraries from four temperatures (69, 63, 58 and 52°C), each representing one of the four main by-temperature clusters identified in UPGMA analysis from Fig. 2, is shown in Table 1. At the highest temperatures, the mats were dominated by 16S rRNA gene sequences similar to the sulfur-oxidizing bacterium *Sulfurihydrogenibium azorense* Az-Fu1 (97–98% identity). The corresponding T-RF (=95) was found at all temperatures examined (Table 1 and Table S1). In addition, another *Aquificales* similar to the sulfur- and hydrogen-oxidizing *Hydrogenobacter* sp. GV1-4 (97% identity, T-RF=125) was detected. The aerobic heterotroph *Thermus kawarayensis* (98% identity, T-RFs=117 and 116) and the anaerobic heterotroph *Eubacterium* sp. (96% identity, T-RF=158) were also observed and widely distributed to lower temperatures.

As the temperature decreased, sequences of photosynthetic anoxygenic bacteria appeared as a dominant component, *i.e.*, *Chloroflexus aggregans* DSM 9485 (97–99% identity, T-RFs= 63 and 69). T-RF=69 was a major component at 63, 58 and 52°C. Two T-RFs (T-RFs=65 and 154), similar to the sulfur-reducing bacterium *Caldimicrobium* sp. were observed, as was the sulfate-reducing *Thermodesulfovibrio* sp. (T-RF=298). Clone libraries indicated that these sulfur reducers were found in small numbers (*i.e.*, *Caldimicrobium*like clones were ~4% of the library at 63°C and *Thermodesulfovibrio*-like clones were ~2% of the libraries at both 63 and 52°C; Table 1). Sequences with low identity to *Chlorobi* (T-RF=83) also appeared; they were distributed to 52°C. The appearance of several members of the *Thermotogae* and *Dictyoglomi*, probable fermenters, also characterized the temperature region around 63°C. One of these, T-RF=265, related to *Fervidobacterium* sp. (62), was detected at 63, 60 and 55°C (Table S1). T-RFLP analysis also detected members of *Deinococcus*/*Thermus* and *Acidobacteria* (T-RFs=123, 148 and 379) below 66°C. In addition, a variety of unidentified T-RFs specific to 62 or 63°C were detected (Table S1).

At 65°C T-RF=490, similar to the oxygenic phototroph “*Thermosynechococcus* (*Synechococcus*) *elongatus*” BP-1 of the *Synechococcus* C1 lineage (63), appeared as an additional primary producer (99% identity); although based on the proportion of the total T-RF signal and clone library analysis, it was not widely distributed above 58°C. This T RF was distributed from 65 to 52°C (Table 1 and S1). A decrease in the population of *Sulfurihydrogenibium* sp. (T-RFs= 95 and 270) and an increase in those of *C. aggregans (*T-RFs=63 and 69) and “*Thermosynechococcus*” (T-RF=490) along the temperature gradient were also shown by changes in the proportional peak intensity of these T-RFs along the gradient (Fig. S2). In addition to the appearance of “*Thermosynechococcus*”, the aerobic heterotroph *Meiothermus* sp. was observed (T-RF=446) at 58°C. From 58°C, several low-identity fragments were identified belonging to the *Aquificales* and *Chloroflexi* (*i.e.*, *Persephonella*-like and *Bellilinia*-like sequences).

The clone library of the sample taken at 52°C indicated the existence of the anoxygenic phototroph *Roseiflexus castenholzii* DSM 13941 (99% identity, T-RFs=61 and 111). T-RF=111 was widely observed at 62, 58, 55 and 52°C by T-RFLP analyses (Table S1). At cooler temperatures (*i.e.*, below 58°C), several additional groups of *Chloroflexi* were detected as a major component of the clone libraries.

### Phylogenetic analysis of *Sulfurihydrogenibium* sp

**Fig. 3:**
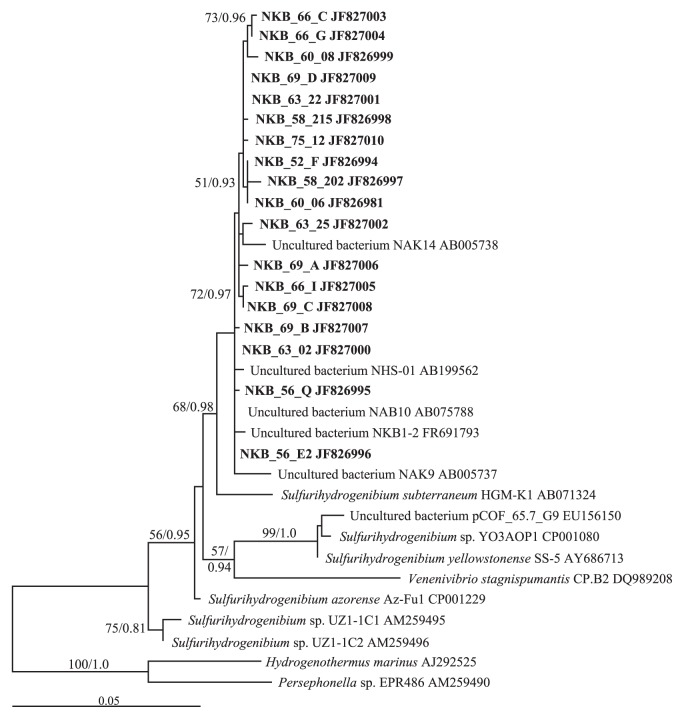
Maximum likelihood phylogenetic tree of *Sulfurihydrogenibium* spp. based on partial 16S rRNA gene sequences. Clones from this study are in bold. Genbank accession numbers follow each sequence name. Bootstrap support >50 for ML and Bayesian posterior probabilities >0.7 are given at the nodes supported by both measures. *Hydrogenothermus marinus* and *Persephonella* sp. EPR486 were used as outgroup taxa.

T-RF=95, related to *Sulfurihydrogenibium* sp., was distributed throughout the temperatures tested. A phylogenetic analysis was performed on clones from Nakabusa with this T-RF. Maximum likelihood and Bayesian analyses were performed on an alignment of 35 taxa that included 18 Nakabusa clones recovered from 8 temperatures in the present study that all shared T-RF=95. The maximum likelihood tree is shown in Fig. 3. All Nakabusa clones from this study clustered together in the phylogeny with other environmental sequences from this spring (NHS-01, NAB10 and NKB1-2) and two sequences (NAK9 and NAK14) from Nakanoyu hot spring, which is approximately 25 km from Nakabusa (40, 43, 57, 81). A second feature of this phylogeny was that all taxa/clones within the tree were grouped together by geographic location. The Nakabusa and Nakanoyu clones plus an additional Japanese isolate (*Sulfurihydrogenibium subterraneum* HGM-K1; from the subsurface hot-aquifer water at Hishikari gold mine) were monophyletic, as were the clones and isolates from North America (YO3AOP1, pCOF_65.7_G9 and SS-5) and East Russia (UZ1-1C1 and UZ1-1C2; 12, 23, 56, 75). The single European isolate *Sulfurihydrogenibium azorense* Az-Fu1 did not affiliate closely with any other group.

## Discussion

Nakabusa hot spring is a promising site to clarify the factors determining microbial community composition and how microbial communities develop, since a spatially heterogeneous environment is observed within areas approximately 1 m in radius. Although numerous studies have characterized the transitions of microbial taxa across temperature gradients at thermal springs (13, 52, 57, 72), only a few have quantitatively analyzed these changes (*e.g.*, 52). Based on their own clone library analyses and the results from several studies for thermal springs worldwide, Skirnisdottir *et al.* (72) observed that, in general community complexity increased inversely with temperature. Using barcoded pyrotag sequencing, Miller *et al.* (52) observed an increase in community complexity with respect to temperature on several hundred meter to kilometer scales. They also proposed that temperature plays a more important role than geography for determining species composition at thermal springs. The present study offers strong support of this idea; here, it was detected that temperature strongly shapes both community assembly and increasing diversity on the centimeter to meter scale. For Nakabusa hot spring, transitions of community composition across different temperatures have been previously observed (43, 57, 73), but the increase of diversity and the structuring of the community along the temperature gradient have not been reported.

Here, the first quantitative analysis of bacterial diversity across a broad temperature gradient (75–52°C) at Nakabusa hot spring revealed a clear relationship between temperature and community composition. Temperature had a clear effect on the number of T-RFs (Fig. 1). Fig. 1 shows strong evidence for an apparently linear temperature effect on diversity; richness increased by roughly 2–3 T-RFs per 5°C decrease. The Shannon diversity index for the individual clone libraries increased inversely to the source temperature of the library (Table 1). Likewise, rarefaction analysis of these libraries indicated that for libraries of comparable sample size, richness estimates were higher in clone libraries from lower temperatures (Fig. S3). In contrast, no clear trends were observed for oxidation-reduction potential (ORP), dissolved oxygen (DO) and sulfide across this temperature gradient (not shown). As would be expected, sulfide concentrations appeared to decline as distance from the source of hot spring waters increased, but the thickness of the mats frequently meant that concentrations of sulfide were maintained within the mats (approximately 0.1 mM).

The cluster analysis shown in Fig. 2 indicates a major break in community composition at 60–62°C, consistent with the previous report of a similar break between 60–66°C in Nakabusa (57). Additional breaks were also observed; in total, four distinct community assemblages were identified related to temperature. The break between the highest temperature zone and the 66 to 60°C zone can be characterized by the appearance of an anoxygenic phototroph, *i.e.*, *Chloroflexus aggregans*. *C. aggregans* provides a second pathway for carbon fixation within the mat from 52 to 66°C in addition to the chemolithotrophic sulfur oxidation performed by members of the *Aquificales*. Furthermore, the filamentous cell morphology and motility of *C. aggregans* may contribute to the structure of the mats as suggested previously (26, 43). We propose that the appearance of *C. aggregans* causes an increase in the productivity and thickness of the mats (~3 mm), and provides a variety of niches for diverse species of anaerobic bacteria (Table 1).

Succession to the next coolest by-temperature community assemblage found from 62 to 55°C may be in part due to the presence of the oxygenic phototroph, related to “*Thermosynechococcus elongatus*” BP-1. This group becomes a major component of the mats at temperatures below 58–60°C (Table 1), but was observed in T-RF profiles up to 65°C (Table S1). This upper range for cyanobacteria in Nakabusa is consistent with previously reported *in situ* pigment analysis by absorbance spectrophotometry, but is higher than previous molecular analyses had reported (57, 73). The ability of cyanobacteria to utilize water as an electron source seems to further increase productivity. Additionally, cyanobacteria produce oxygen and extracellular organic compounds which may allow for the association of a variety of heterotrophs, including several species of aerobic bacteria, to exist within the thick mats (Table 1). Temperatures below 55°C were characterized by the further appearance of a variety of heterotrophs. In this way, transitions to cooler communities were largely characterized by the successive addition of chemolithotrophs, anoxygenic phototrophs, and oxygenic phototrophs (Table 1, Table S1 and Fig. S2). Based on previously reported genomic information, these transitions also indicated the successive addition of carbon fixation pathways, *i.e.*, the reductive TCA cycle by *Sulfurihydrogenibium* (68), 3-hydroxypropionate pathway by *Chloroflexus* (41), and reductive pentose phosphate cycle by “*Thermosynechococcus elongatus*” (58). This addition of different autotrophic pathways provides a diversity of metabolic intermediates and expands the overall ability of the community to fix carbon under different environmental conditions. These factors may combine to expand available niches in the mats and to increase the matter and energy flux in the system, thus facilitating the observed increase in diversity and biomass. In natural ecosystems, it has been reported that productivity has a positive linear relationship with biodiversity on a local scale (16). These results strongly support this idea. Possible mechanisms of diversification concomitant with productivity are as follows: high productivity thickens the mat, the thick mat structure provides additional niches, and multiple primary producers lead to the diversification of available organic compounds. Furthermore, such biomass may allow for the presence in the mat community of bacteria (T-RFs=446 and 148) similar to proteolytic bacteria that produce cell-lytic enzymes (59, 85).

*Ex situ* anaerobic incubation of mats collected in Nakabusa from 75 to 65°C have shown biological sulfide production (43, 57). In addition to members of the *Thermodesulfobacteria*, (T-RFs=65, 154), which were previously identified as potential sulfide producers in Nakabusa (57), the clone library at 63°C also identified T-RF=298 as a candidate sulfate-reducing bacteria. These T-RFs were also detected at lower temperatures (Table S1). An anaerobic niche which is required for sulfate-reducing bacteria was likely provided by aerobic respiration of *Sulfrihydrogenibium* within the mats at 63°C. The sulfide produced by sulfate reducers may be simultaneously oxidized to sulfate and elemental sulfur (S^0^) by aerobic chemolithotrophy of *Sulfrihydrogenibium* and anaerobic photoautotrophy of *C. aggregans*, respectively, as suggested by Kubo *et al.* (43).

There was evidence of endemism in Japan for *Sulfurihydrogenibium* (Fig. 3), with all clones from Nakabusa, including clones NHS-01, NAB10 and NKB1-2 from previous studies clustering together (40, 43, 57). Such geographic diversification is of great interest to elucidate the ecological roles and diversification of this group. In contrast to an earlier finding that *Aquificales* were only present from 66°C and above (57), these results also indicate that *Sulfurihydrogenibium* sp. (T-RF=95) was present throughout the temperature gradient (Table 1 and Table S1). Kimura *et al.* (39) reported the high productivity of microbial mats dominated by *Sulfurihydrogenibium* sp. at Nakabusa hot spring. Continuous supply of electron donors from hot spring water may support the growth of *Sulfurihydrogenibium* sp. in a wide range of temperatures. Previous reports have revealed different temperature optima for isolated strains and divergence in gene sequences in this group across thermal gradients (23, 67). Takacs-Vesbach *et al.* has shown that in Yellowstone National Park this genus has a biogeography defined by different calderas (74). In Nakabusa hot spring, phylogenetic analysis of sequences belonging to T-RF=95 showed no evidence of structuring by temperature (Fig. 3). The conserved nature of the 16S rRNA locus may obscure the real relationships between temperature and *Sulfurihydrogenibium* diversity. Genetic and physiological identification of locally adapted ecotypes of *Sulfurihydrogenibium* in the narrow area at Nakabusa hot spring (Fig. 3) is worthy of further investigation.

Here it is shown that the microbial community at Nakabusa hot spring contains an amazing diversity; 13 bacterial phyla were identified across a temperature range of 23°C. In comparison, insufficient DNA fragments were recovered for *Archaea*, even when using targeted primers. This suggests that the archaeal population was much lower than that of *Bacteria*. From the deeply branching bacterial phylum *Aquificales* to more recently diverged photoheterotrophic and photoautotrophic bacteria, the successive appearance of a diverse species of bacteria was characterized by the addition of species rather than by the replacement of species (Table 1 and Table S1). The decrease in temperature allowed an increasing variety of bacterial species to grow; however, bacteria also appeared to be able to co-exist with the bacterial members proliferating from higher temperatures. Elucidation of the spatial and metabolic relationships between these groups inevitably gives us a deeper insight into the development and structuring of microbial ecosystems.

## Supplementary Material



## Figures and Tables

**Table 1:** Clone number and Shannon diversity index at different temperatures

T-RF (bp)	No. of clones				Accession no.	Closest cultured match and identity	Putative phylum or class	Select predicted metabolic characteristics (reference)

	52°C	58°C	63°C	69°C				
95^a^	1	2	7	35	JF826981	*Sulfurihydrogenibium azorense* Az-Fu1 97%	*Aquificales*	Sulfur/H_2_ oxidation (2)
125			1	1	JF826993	*Hydrogenobacter* sp. GV1-4 97%	*Aquificales*	Sulfur/H_2_ oxidation (38)
117		2		3	JF826970	*Thermus kawarayensis* 98%	*Deinococcus/Thermus*	Aerobic heterotroph (45)
116		1			JF82690	*Thermus kawarayensis* 98%	*Deinococcus/Thermus*	Aerobic heterotroph (45)
158		1	1	3	JF826973	*Eubacterium* sp. (OS type L) 96%	*Firmicutes*	Fermentation (77)
557	1			1	JF827016	*Caldisericum exile* AZM16c01 81%	*Caldiserica*	Fermentation (54)
262				1	JF827015	*Fervidobacterium* sp. YNP 98%	*Thermotogae*	Fermentation (32)
453			4		JF827017	*Fervidobacterium nodosum* Rt17-B1 98%	*Thermotogae*	Fermentation (32)
265			1		JF826986	*Fervidobacterium riparium* 1445t 99%	*Thermotogae*	Fermentation (32)
118			1		JF826983	*Thermotogales* sp. SRI-15 95%	*Thermotogae*	Fermentation (66)
300			7		JF826982	*Dictyoglomus* sp. 1512 98%	*Dictyoglomi*	Fermentation (42)
69	7	7	16		JF826969	*Chloroflexus aggregans* DSM 9485 98%	*Chloroflexi*	Anoxygenic phototroph (25)
63		1	3		JF826984	*Chloroflexus aggregans* DSM 9485 98%	*Chloroflexi*	Anoxygenic phototroph (25)
83	1	3	2		JF826976	*Ignavibacterium album* 86%	*Chlorobi*	Fermentation (35)
65			1		JF826988	*Caldimicrobium rimae* 95%	*Thermodesulfobacteria*	Sulfur reduction (53)
154			1		JF826985	*Caldimicrobium rimae* 95%	*Thermodesulfobacteria*	Sulfur reduction (53)
298	1		1		JF826987	*Thermodesulfovibrio hydrogeniphilus* Hbr5 94%	*Nitrospirae*	Sulfate reduction (28)
490	9	4			JF826965	*Thermosynechococcus elongatus* BP-1 99%	*Cyanobacteria*	Oxygenic phototroph (29)
446		5			JF826977	*Meiothermus* sp. L462 100%	*Deinococcus/Thermus*	Aerobic heterotroph (85)
148	2	1			JF826974	*Meiothermus* sp. L462 95%	*Deinococcus/Thermus*	Aerobic heterotroph (85)
145		1			JF827014	*Acidobacteria bacterium* KBS 96 93%	*Acidobacteria*	Aerobic heterotroph (11)
141		1			JF826972	*Persephonella* sp. 124-5-R1-1 85%	*Aquificales*	H_2_ oxidation (19)
191		1			JF826979	*Bellilinea caldifistulae* 89%	*Chloroflexi*	Fermentation (80)
513		3			JF826978	*Bellilinea caldifistulae* 90%	*Chloroflexi*	Fermentation (80)
111	3				JF826967	*Roseiflexus castenholzii* DSM 13941 99%	*Chloroflexi*	Anoxygenic phototroph (27)
61	1				JF827013	*Roseiflexus castenholzii* DSM 13941 99%	*Chloroflexi*	Anoxygenic phototroph (27)
109	5				JF826964	*Chloroflexi* bacterium GNS-1 85%	*Chloroflexi*	Fermentation (21)
426	3				JF827011	*Dehalococcoides* sp. 11a5 85%	*Chloroflexi*	Anaerobic H_2_ oxidation (50)
173	2				JF827012	*Elioraea tepidiphila* TU-7 96%	*Alphaproteobacteria*	Aerobic heterotroph (1)
77	2				JF826966	*Thiobacillus aquaesulis* 92%	*Betaproteobacteria*	Sulfur oxidation (79)
450	1				JF826968	*Alcaligenaceae* bacterium BL-169 95%	*Betaproteobacteria*	Aerobic heterotroph (5)
Total	39	33	46	44				
b*H′*	2.52	2.39	1.99	0.81				

a Several sequences for this T-RF are also listed in Fig. 3,

b Shannon diversity index for 52°C includes clones for 3 unidentified TRFs=107, 156 and 191.
