# Supplementary material for: Diversification of Bacterial Community Composition along a Temperature Gradient at a Thermal Spring
Source: Microbes Environ. 2012 May 17;27(4):374–81. doi: 10.1264/jsme2.ME11350 (PMC4103544; doi:10.1264/jsme2.ME11350)
Supplement: Supplementary file 1 [file 27_374_s1.pdf]

Supplementary table and figures (R. Craig Everroad)

Table S1. Distribution of environmental T-RFs sorted by highest temperature of appearance and representative clone

Fig. S1. Nakabusa hot spring mats and locations sampled.

Fig. S2. Proportion of total T-RF signal at each temperature related to *Sulfurihydrogenibium* sp., *Chloroflexus aggregans*, "*Thermosynechococcus*" sp., and polynomial fit regression lines. (n = 14).

Fig. S3. Rarefaction curves for taxa (T-RF of clones) for clone libraries from 52, 58, 63 and 69˚C.

Table S1. Distribution of environmental T-RFs sorted by highest temperature of appearance and representative clone

T-RF	Temperature (˚C)														Representative			
(bp)	52	55	56	58	60	62	63	65	66	67	69	70	71	75	clone[a]	Accession no.	Closest cultured match and ID	Putative phylum
95	+	+	+	+	+	+	+	+	+	+	+	+	+	+	NKB_60_06[b]	JF826981	*Sulfurihydrogenibium azorense* Az-Fu1 97%	*Aquificales*
270			+		+		+	+	+	+	+	+	+	+	NKB_66_N	JF826990	*Sulfurihydrogenibium azorense* Az-Fu1 98%	*Aquificales*
117	+	+	+	+		+				+	+	+	+	+	NKB_56_F	JF826970	*Thermus kawarayensis* 98%	*Deinococcus/Thermus*
88													+					
141		+	+					+	+	+	+	+			NKB_56_V	JF826972	*Persephonella* sp. 124-5-R1-1 85%	*Aquificales*
116		+	+	+	+	+			+	+	+	+			NKB_58_047	JF826980	*Thermus kawarayensis* 98%	*Deinococcus/Thermus*
158	+	+	+	+		+			+			+			NKB_56_N2	JF826973	*Eubacterium* sp. (OS type L) 96%	*Firmicutes*
159		+	+						+	+	+							
125	+								+	+	+				NKB_69_H2	JF826993	*Hydrogenobacter* sp. GV1-4 97%	*Aquificales*
282		+	+							+	+				NKB_66_U	JF826991	*Hydrogenobacter thermophilus* TK-6 94%	*Aquificales*
83	+	+		+	+	+	+	+	+		+				NKB_56_U2	JF826976	*Ignavibacterium album* 86%	*Chlorobi*
135	+	+							+		+				NKB_66_H	JF826989	*Eubacterium* sp. (OS type L) 95%	*Firmicutes*
280									+		+							
154	+	+					+				+				NKB_63_33	JF826985	*Caldimicrobium rimae* 95%	*Thermodesulfobacteria*
96											+							
575											+							
69	+	+	+	+	+	+	+	+	+						NKB_56_C	JF826969	*Chloroflexus aggregans* DSM 9485 98%	*Chloroflexi*
118	+				+		+	+	+						NKB_63_05	JF826983	*Thermotogales* sp. SRI-15 95%	*Thermotogae*
379						+		+	+						NKB_56_T2	JF826975	*Rhodothermus* sp. PRI2902 81%	*Deinococcus/Thermus*
123	+	+	+	+		+			+						NKB_56_I	JF826971	*Acidobacteriaceae* bacterium Gsoil 1619 87%	*Acidobacteria*
148	+	+	+	+		+			+						NKB_56_O2	JF826974	*Meiothermus* sp. L462 95%	*Deinococcus/Thermus*
283									+									
490	+	+		+	+			+							NKB_52_C	JF826965	*Thermosynechococcus elongatus* 99%	*Cyanobacteria*
67					+	+	+											
63	+	+		+		+	+								NKB_63_10	JF826984	*Chloroflexus aggregans* DSM 9485 98%	*Chloroflexi*
298	+	+				+	+								NKB_63_50	JF826987	*Thermodesulfovibrio hydrogeniphilus* Hbr5 94%	*Nitrospirae*
265		+			+		+								NKB_63_38	JF826986	*Fervidobacterium riparium* 1445t 99%	*Thermotogae*
65	+	+					+								NKB_63_56	JF826988	*Caldimicrobium rimae* 95%	*Thermodesulfobacteria*
300							+								NKB_63_01	JF826982	*Dictyoglomus* sp. 1512 98%	*Dictyoglomi*
107							+											
177							+											
180							+											
189							+											
302							+											
369							+											
454							+											
111	+	+		+		+									NKB_52_Z	JF826967	*Roseiflexus castenholzii* DSM 13941 99%	*Chloroflexi*
85	+	+				+												
201	+					+												
77	+					+									NKB_52_Q	JF826966	*Thiobacillus aquaesulis* 92%	*Proteobacteria* (β)
70						+												
274						+												
460						+												
491						+												
518						+												
486				+	+													
513				+	+										NKB_58_010	JF826978	*Bellilinea caldifistulae* 90%	*Chloroflexi*
447	+	+			+													
450					+										NKB_52_E2	JF826968	*Alcaligenaceae* bacterium BL-169 95%	*Proteobacteria* (β)
207			+	+														
446				+											NKB_58_001	JF826977	*Meiothermus* sp. L462 100%	*Deinococcus/Thermus*
448				+														
82		+	+															
109	+	+													NKB_52_A	JF826964	*Chloroflexi* bacterium GNS-1 85%	*Chloroflexi*
478	+	+																
483		+													NKB_66_I2	JF826992	*Rhodothermus* sp. Ae70-7C-S 86%	*Deinococcus/Thermus*
78		+																
168		+																
171		+																
511		+																
191	+														NKB_58_031	JF826979	*Bellilinea caldifistulae* 89%	*Chloroflexi*
86	+																	
156	+																	
484	+																	
510	+																	
521	+																	

[a]First number in clone name indicates source temperature (˚C)

[b]Several sequences for this T-RF are also listed in Figure 3

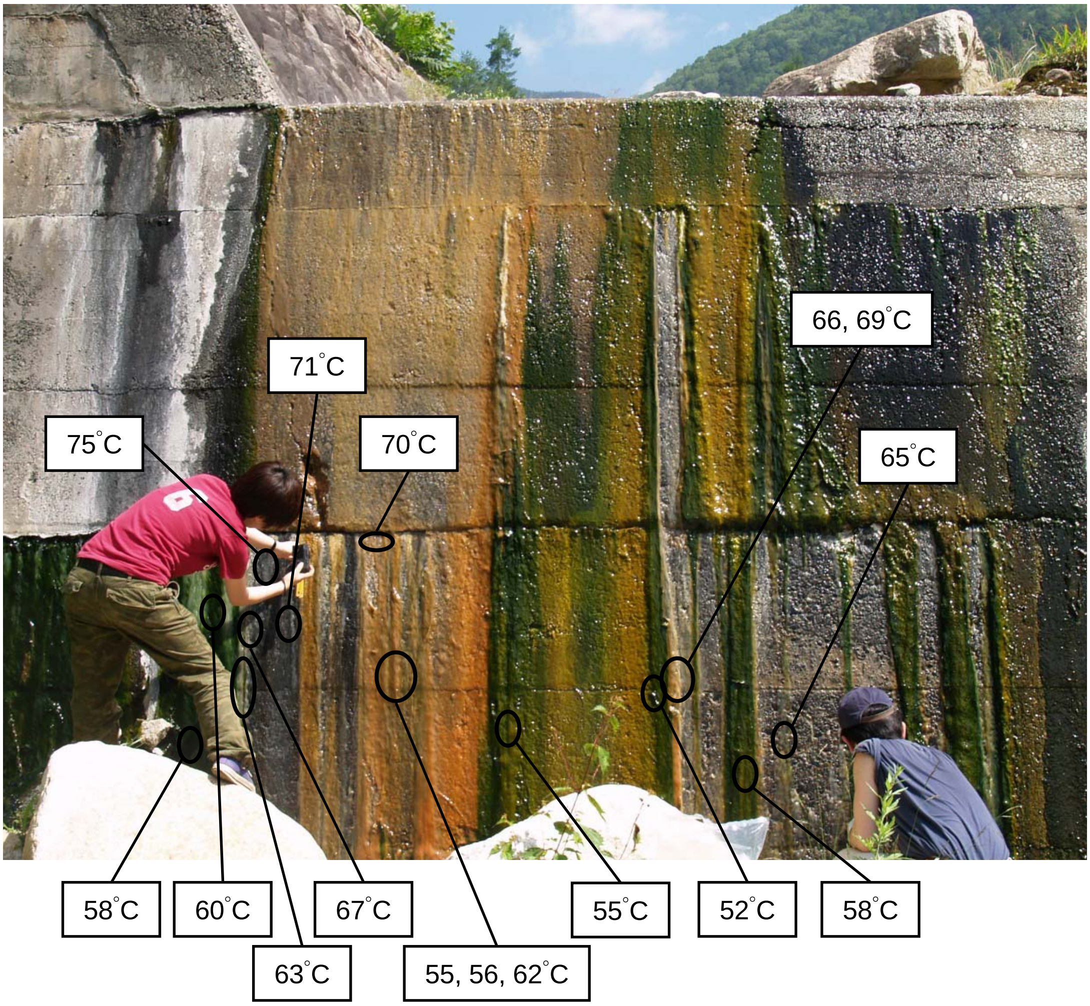

Everroad et al. Fig. S1

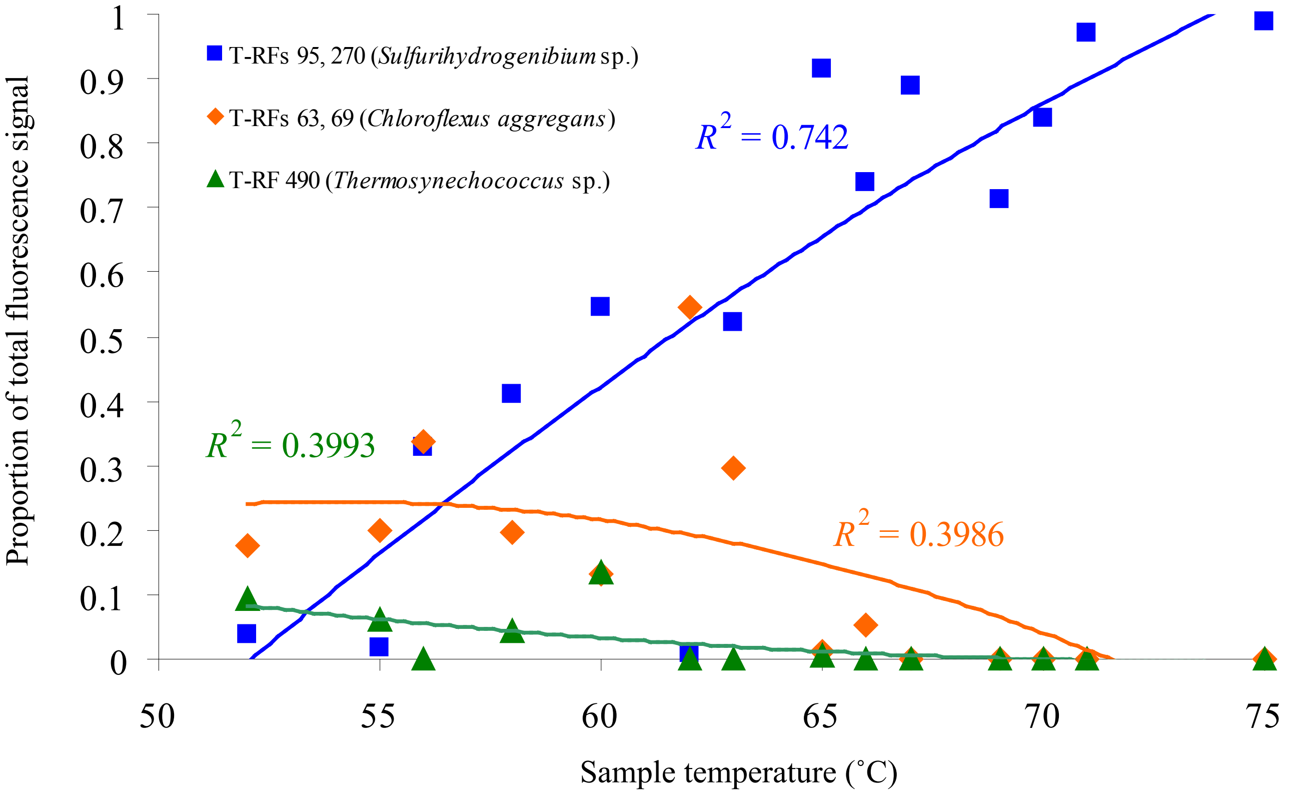

Fig. S2 Everroad et al.

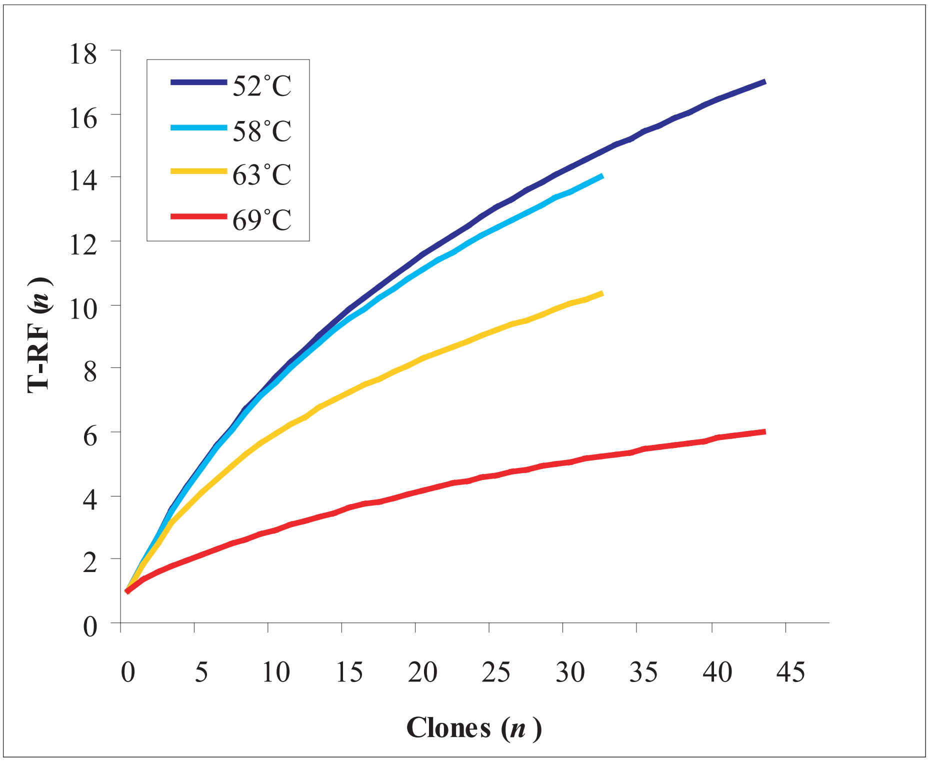

Fig. S3 Everroad et al.
